# Clinical characteristics of severe influenza as a risk factor for febrile seizures in children: a retrospective analysis

**DOI:** 10.3389/fped.2024.1418499

**Published:** 2024-10-23

**Authors:** Peng Li, Mei Chen, Daobin Wang, Xue Zhang, Ruiyang Sun, Wanyu Jia, Shuqin Fu, Junhao Cui, Chunlan Song

**Affiliations:** ^1^ Pediatric Emergency Department, Henan Children's Hospital Zhengzhou Children's Hospital, Children's Hospital Affiliated to Zhengzhou University, Zhengzhou, Henan Province, China; ^2^ Pediatric Emergency Department, Henan Provincial Engineering Research Center for Diagnosis and Treatment of Pediatric Infections and Critical Illnesses, Zhengzhou, Henan Province, China; ^3^ Pediatric Department, Zhecheng County People's Hospital, Shangqiu, Henan Province, China

**Keywords:** influenza, febrile seizures, risk factors, receiver operating characteristic curve, multivariate logistic regression

## Abstract

**Objective:**

To retrospectively analyze the clinical characteristics and independent risk factors of severe influenza combined with febrile seizures, and to provide more basis for early clinical intervention.

**Methods:**

A total of 161 children with severe influenza were collected as study subjects and divided into convulsive (FC) group (40 cases) and non-convulsive (NFC) group (121 cases) according to whether they had febrile seizures. The demographic characteristics and clinical data of the two groups were analyzed. Multivariate logistic regression was used to analyze the risk factors of severe influenza combined with febrile seizures. The predictive efficacy was evaluated by receiver operating characteristic (ROC) curve analysis.

**Results:**

(1) Multiple logistic regression analysis revealed that C-reactive protein (CRP) levels, Serum interleukin 6 (IL-6) levels, Days from onset of Flu symptoms to hospitalization, cerebrospinal fluid protein (CSF-TP) levels and the influenza virus type A (FluA) infection rate were found to be independent risk factors for severe influenza combined with febrile seizures in children. (2) ROC curve analysis showed that the cut-off value of CRP, Serum IL-6, Days from onset of Flu symptoms to hospitalization and CSF-TP were 7.57 mg/L, 9.84 pg/ml, 4.5 days and 194.8 mg/L, respectively.

**Conclusion:**

Children with severe influenza with CRP ≥ 7.57 mg/L, Serum IL-6 ≥ 9.84 pg/ml, Days from onset of Flu symptoms to hospitalization ≤4.5 days, CSF-TP ≥ 194.8 mg/L and FluA had a significantly increased risk of febrile seizures. It is useful for clinicians to determine the risk of severe influenza combined with febrile seizures, to adjust the early treatment plan, and to reduce the incidence of critically ill patients.

## Introduction

Influenza is an acute respiratory infectious disease that is prone to pandemics and outbreaks because of its high incidence and rapid spread (1). Influenza is characterized by clinical symptoms such as fever, myalgia, and headache and may be accompanied by comorbidities in various systems, such as the respiratory, digestive, and neurological systems (2). Children are susceptible to influenza and are at high risk of severe influenza (3), which may cause multiple-organ dysfunction and threaten life. Children with severe influenza combined with febrile seizures may suffer irreversible neurological damage because of recurrent or prolonged convulsive seizures, however the pathogenesis of influenza combined with febrile seizures is unclear. Therefore, early detection of high-risk factors for influenza combined with febrile seizures and timely targeted treatment are important for minimizing neurological damage. At present, studies on the risk factor screening of influenza complicated with febrile seizures are rare. These studies are based mainly on clinical experience, and the related scientific evidence is insufficient.

In this study, we retrospectively analyzed the clinical characteristics and laboratory parameters of 161 children with severe influenza combined with febrile seizures, to investigate the independent risk factors of severe influenza combined with febrile seizures in children and to provide more clinical basis.

## Data and methods

Patients: A total of 161 children with severe influenza admitted to Henan Provincial Children's Hospital from October 2018 to October 2020 were selected as the study objects, and clinical data were retrieved from the electronic medical records system of Henan Provincial Children's Hospital. This study was approved by the Medical Ethics Committee of Henan Children's Hospital (Review number: 2022-K-L046).

The inclusion criteria for severe influenza, were as follows: (1) aged 0–14 years, (2) positive influenza A/B antigen by immunochromatographic colloidal gold assay or positive influenza A/B viral RNA by reverse transcription polymerase chain reaction assay, (3) diagnosis and treatment of a severe case, based on the diagnostic criteria in the “Influenza Diagnosis and Treatment Protocol (2020 Edition) China” (4), and one of the following conditions: ① persistent high fever >3 days, accompanied by coughing, chest pain etc. ② Dyspnea, lip cyanosis. ③ Disturbance of consciousness, convulsions, etc. ④ Emesis, diarrhea, etc. ⑤ Complicated with pneumonia, ⑥ worsening of underlying diseases, or ⑦ other clinical conditions that require hospitalization.

The inclusion criteria for febrile seizures were as follows: (1) met the criteria for febrile seizures defined by the American Academy of Pediatrics (5). (2) Has no abnormality in the head upon imaging.

The exclusion criteria for patients were as follows: (1) infection caused by other pathogens or co-infecion with other pathogens, (2) incomplete clinical data, or (3) had a previous history of epilepsy and brain trauma.

### Clinical data collection

The general information collected included sex, age, influenza virus type, days from onset of Flu symptoms to hospitalization, duration of hospital stay and vomiting, etc.

Laboratory data: antigen/RNA positivity for influenza virus type A (FluA) or influenza virus type B (FluB). The white blood cell (WBC) count, neutrophil count (NE), lymphocyte count (LY), platelet count (PLT), C-reactive protein (CRP), interleukin 6 (IL-6), procalcitonin (PCT), immunoglobulin E (IgE), immunoglobulin G (IgG), immunoglobulin A (IgA), immunoglobulin M (IgM), complement C3 (C3), complement C4 (C4), T lymphocytes (T), T8 lymphocytes (T8), T4 lymphocytes (T4), T4/T8, NK cells (NK), B cells (B), glutamic-pyruvic transaminase (ALT), glutamic oxalacetic transaminase (AST), lactate dehydrogenase (LDH), creatinine (CR), urea, glucose (GLU), creatine kinase (CK), creatine kinase isoenzyme (CK-MB), B-type natriuretic peptide (BNP), neuron-specific enolase (NSE), S100β protein (S100β), cerebrospinal fluid white blood cell (CSF-WBC), cerebrospinal fluid chloride (CSF-CL), cerebrospinal fluid protein (CSF-TP), and cerebrospinal fluid glucose (CSF-Glu) were examined for the first time after admission.

### Statistical analysis

Statistical analysis was performed via SPSS 26.0 statistical software, and the measurement data are expressed as the means ± standard deviations (*x* ± *s*) and were analyzed via t test. The count data are expressed as cases (constitutive ratios) and were analyzed via the *χ*^2^ test. Variables with statistically significant differences according to the unit regression analysis were included in the multiple logistic regression analysis to identify independent risk factors with diagnostic value. Receiver operating characteristic (ROC) curves were used to evaluate the efficacy of the study indicators. *P* < 0.05 was considered statistically significant.

## Results

### Clinical characteristics

This study included 161 children with severe influenza, who were divided into a convulsive (FC) group (40 cases) and a non-convulsive (NFC) group (121 cases). Univariate analysis of the two groups revealed that children with severe influenza in the FC group had fewer days from onset of Flu symptoms to hospitalization (*t* = 6.29). Compared with the NFC group, the FC group presented a greater incidence of headache (*x*^2^ = 11.14, OR = 6.39, 95% CI: 2.15–18.98), FluA (*x*^2^ = 4.23, OR = 0.27, 95% CI: 0.07–0.94), and disturbance of consciousness (*x*^2^ = 4.84, OR = 2.27, 95% CI: 1.04–4.71). The incidence of pneumonia (*x*^2^ = 10.49, OR = 0.24, 95% CI = 0.10–0.57) was lower in the FC group than in the NFC group (Table 1).

**Table 1:** Comparison of clinical characteristics between the two groups.

Variables	FC group (*n* = 40)	NFC group (*n* = 121)	*x*^2^/*t*	*P*	OR [95% confidence interval (CI)]
Male (*n*, %)	23, 57.50	83, 68.60	1.63	0.20	1.61 (0.77–3.37)
Age (months)	39.90 ± 37.28	38.45 ± 30.54	0.25	0.81	
Fever >3 days (*n*, %)	4, 10.00	19, 15.70	0.79	0.38	1.68 (0.53–5.26)
Emesis (*n*, %)	0, 0.00	15, 12.40	2.74	0.09	8.69 (0.67–112.18)
Diarrhea (*n*, %)	1, 2.50	9, 7.44	1.13	0.29	3.13 (0.39–25.53)
Flu type					
FluA (*n*, %)	37, 92.50	93, 76.86	4.23	0.04	0.27 (0.07–0.94)
FluB (*n*, %)	2, 5.00	23, 19.01	3.46	0.06	0.14 (0.02–1.11)
FluA + B (*n*, %)	1, 2.50	5, 4.13	0.73	0.39	3.60 (0.19–68.34)
Symptoms					
Pneumonia (*n*, %)	14, 35.00	84, 69.42	10.49	0.00	0.24 (0.10–0.57)
Headache (*n*, %)	10, 25.00	6, 4.96	11.14	0.00	6.39 (2.15–18.98
Disturbance of consciousness (*n*, %)	20, 50.00	37, 30.58	4.84	0.03	2.27 (1.04–4.71)
Irritable (*n*, %)	2, 5.00	1, 0.83	2.21	0.13	6.32 (0.56–71.60)
Days from onset of Flu symptoms to hospitalization (days)	1.70 ± 1.16	4.30 ± 2.52	−6.29	0.00	
Duration of hospital stay (days)	5.00 ± 3.39	4.27 ± 3.74	1.09	0.28	

### Laboratory data

According to the laboratory data, the PLT level in the FC group was lower than that in the NFC group (*t* = 2.34, *P* < 0.05 for all), and the levels of CRP, serum IL-6, C4, C3, and CSF-TP were greater than those in the NFC group (*t* = 2.22, 2.26, 3.31, 2.75, 5.39, *P* < 0.05) (Table 2).

**Table 2:** Comparison of laboratory data between the two groups.

Variables	FC group (*n* = 40)	NFC group (*n* = 121)	*t*	*P*
Blood routine				
WBC (×10^9^/L)	5.00 ± 1.98	7.84 ± 3.74	−1.97	0.055
NE (×10^9^/L)	2.72 ± 1.47	5.42 ± 3.25	−2.00	0.050
LY (×10^9^/L)	1.98 ± 1.08	2.17 ± 2.05	−0.22	0.83
PLT (×10^9^/L)	181.14 ± 63.66	277.83 ± 106.86	−2.34	0.02
CRP (mg/L)	14.15 ± 11.75	8.48 ± 14.69	2.22	0.03
IL-6 (pg/ml)	120.47 ± 199.24	36.31 ± 205.65	2.26	0.03
PCT (ng/ml)	0.23 ± 0.23	1.60 ± 8.75	−0.41	0.68
Immune function				
IgE (g/L)	210.33 ± 216.08	377.65 ± 681.51	−0.49	0.63
IgG (g/L)	7.28 ± 0.96	7.88 ± 4.68	−0.33	0.74
IgA (g/L)	0.68 ± 0.39	7.88 ± 4.68	−1.41	0.16
IgM (g/L)	0.83 ± 0.24	0.97 ± 0.47	−0.78	0.44
C3 (g/L)	1.03 ± 0.23	0.84 ± 0.41	2.75	0.01
C4 (g/L)	11.87 ± 28.64	0.25 ± 0.08	3.31	0.00
T (%)	60.91 ± 9.67	56.75 ± 9.64	1.08	0.28
T8 (%)	22.23 ± 6.35	21.06 ± 7.37	0.40	0.69
T4 (%)	35.67 ± 11.94	31.72 ± 9.43	1.02	0.31
T4/T8	1.83 ± 1.13	1.71 ± 0.81	0.35	0.73
NK (%)	12.01 ± 7.38	14.23 ± 8.66	−0.65	0.52
B (%)	25.44 ± 6.87	27.25 ± 10.49	−0.44	0.66
Blood biochemistry				
ALT (U/L)	34.01 ± 5.67	31.64 ± 25.77	0.24	0.81
AST (U/L)	49.33 ± 16.32	50.98 ± 48.76	−0.09	0.93
LDH (U/L)	248.68 ± 121.89	316.93 ± 284.72	−0.58	0.56
CR (umol/L)	23.61 ± 5.23	27.61 ± 9.73	−1.06	0.29
Urea (mmol/L)	3.55 ± 1.93	3.39 ± 1.29	0.32	0.75
GLU (mmol/L)	5.69 ± 0.84	6.27 ± 1.25	1.19	0.24
CK (U/L)	256.53 ± 303.32	355.65 ± 1,848.54	−0.13	0.89
CK-MB (U/L)	28.40 ± 10.84	49.24 ± 173.15	−0.29	0.77
BNP (pg/ml)	437.45 ± 613.41	455.20 ± 619.51	−0.07	0.94
NSE (ng/ml)	7.99 ± 1.16	6.87 ± 6.98	0.22	0.83
S100β (pg/ml)	0.75 ± 1.16	0.34 ± 0.69	1.17	0.24
Cerebrospinal fluid biochemistry				
CSF-WBC (×10^9^/L)	0.003 ± 0.003	0.004 ± 0.009	−0.35	0.73
CSF-CL (mmol/L)	118.80 ± 2.39	120.88 ± 2.76	−1.59	0.12
CSF-TP (mg/L)	248.69 ± 129.25	172.69 ± 49.60	5.39	0.00
CSF-Glu (mmol/L)	3.8 ± 0.45	3.72 ± 0.92	0.19	0.85

### Multivariate logistic regression

The variables related to univariate analysis were defined, assigned and included in the multiple logistic regression model for analysis. CRP (*x*^2^ = 5.19, OR = 1.04, 95% CI = 1.01–1.07), serum IL-6 (*x*^2^ = 4.13, OR = 1.00, 95% CI = 1.00–1.01), days from onset of Flu symptoms to hospitalization (*x*^2^ = 21.60, OR = 0.48, 95% CI = 0.35–0.65), CSF-TP (*x*^2^ = 7.50, OR = 1.01, 95% CI = 1.01–1.03), and FluA (*x*^2^ = 4.51, OR = 4.51, 95% CI = 1.12–18.10) were found to be independent risk factors for severe influenza and febrile seizures in children (*P* < 0.05) (Table 3).

**Table 3:** Results of multiple logistic regression analysis of the two groups.

Variables	*Β*	*x* ^2^	SE	*P*	OR [95% confidence interval (CI)]
CRP	0.04	5.19	0.02	0.02	1.04 (1.01–1.07)
IL-6	0.002	4.13	0.001	0.04	1.00 (1.00–1.01)
Days from onset of Flu symptoms to hospitalization	−0.73	21.60	0.16	0.00	0.48 (0.35–0.65)
CSF-TP	0.01	7.50	0.01	0.01	1.01 (1.01–1.03)
FluA	1.51	4.51	0.71	0.03	4.51 (1.12–18.10)

### ROC curve evaluation for multi-index combined diagnosis

**Figure 1:**
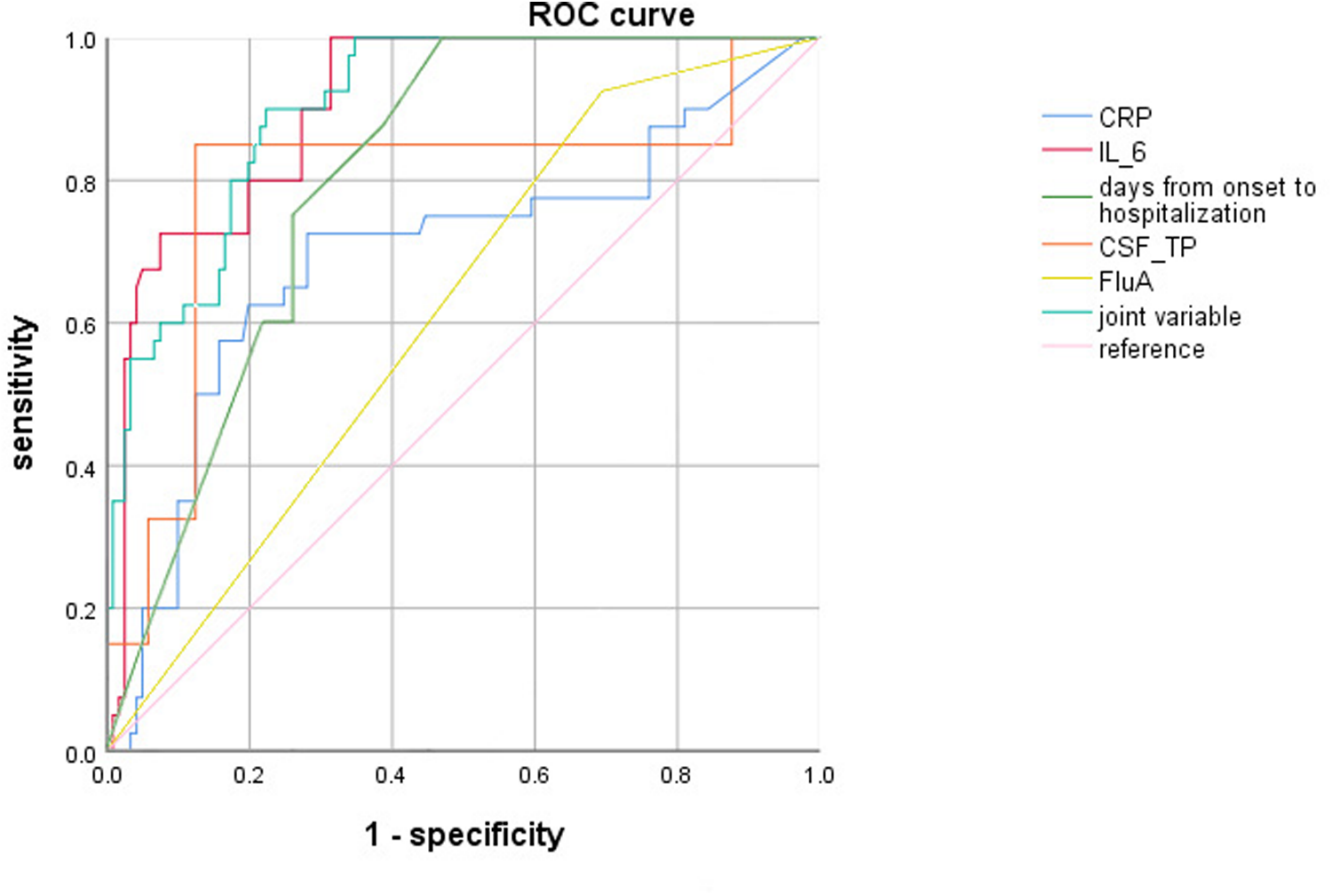
ROC curve for the five indicators combined.

ROC curve analysis revealed showed that CRP ≥ 7.57 mg/L, Serum IL-6 ≥ 9.84 pg/ml, days from onset of Flu symptoms to hospitalization ≤4.5 days, CSF-TP ≥ 194.80 mg/L and FluA increased the risk of severe influenza combined with febrile seizures. In addition, combining the above indicators further improved the efficiency [AUC (95% CI) = 0.903 (0.86–0.95)], and the sensitivity and specificity were 0.90 and 0.78, respectively (Table 4, Figure 1).

**Table 4:** The results of ROC curve evaluation multi-index combined diagnosis.

Variables	AUC	Cut-off value	Youden index	Sensitivity	Specificity	95% CI
CRP	0.696	7.57	0.44	0.73	0.72	(0.59–0.80)
IL-6	0.905	9.84	0.69	1.00	0.69	(0.86–0.95)
Days from onset of Flu symptoms to hospitalization	0.806	4.50	0.54	1.00	0.54	(0.74–0.87)
CSF-TP	0.793	194.80	0.73	0.85	0.88	(0.70–0.89)
FluA	0.615	/	0.23	0.93	0.31	(0.52–0.71)
Joint variable	0.903	/	0.68	0.90	0.78	(0.86–0.95)

## Discussion

Influenza is an acute respiratory infectious disease caused by the influenza virus. The influenza viruses that infect humans are mainly type A and type B. This disease is highly contagious and can cause influenza pandemics or worldwide outbreaks. Children are at high risk for influenza, and some studies have shown that during high flu seasons, the infection rate can reach 50% (6, 7). Influenza virus can cause a variety of neurological complications, such as febrile seizures, encephalopathy, acute necrotizing encephalopathy, and so on, but the most common complication is febrile seizures (8, 9). Influenza A virus is more likely to cause febrile seizures than other respiratory viruses are, which is related to a new amino acid substitution in the receptor binding site of the hemagglutinin gene of influenza A virus, which makes influenza A virus more neurotropic to the brain (10). The present study revealed that Flu A was more likely to induce febrile seizures, as in the aforementioned study. A multicenter study revealed that 38.7% of children with influenza combined with neurological symptoms had one or no respiratory symptoms (11). Several studies have reported that severe influenza requiring mechanical ventilation is mostly due to central respiratory failure, and these children have milder lung infections (12). In this study, the incidence of pneumonia in the FC group was significantly lower than that in the NFC group, indicating that pulmonary infection in children with severe influenza complicated with febrile seizures was not serious or there was no pulmonary infection. The incidence of febrile seizures was unrelated to the occurrence and severity of fever, cough or other symptoms, which was the same as that reported in the above studies. In this study, the mean number of days from onset of Flu symptoms to hospitalization in the FC group was 1.70 days, which is close to the 2–4 days reported in some studies (13). The PLT and PCT levels in the NFC group were significantly higher than that in the FC group, and the average days from onset to admission in the NFC group was 4.30 days, which was considered to be related to mixed bacterial infection in the later stage, which is consistent with the findings of previous studies (14).

The role of inflammatory mediators as febrile trigger factors in febrile seizures has been explored clinically. Several studies have shown that certain inflammatory responses occur in the body after infection with Flu (15). Inflammation indicators include CRP and IL-6. CRP is an acute-phase reactant of proinflammatory cytokines released by the body after infection. Another study revealed a significantly greater incidence of severe influenza in children with a CRP > 8 mg/L and concluded that elevated CRP levels correlate with disease severity (16–18). The present study revealed that the CRP level in the FC group was significantly greater than that in the NFC group, demonstrating that CRP is an important inflammatory indicator of severe influenza combined with febrile seizures.

IL-6 is a proinflammatory cytokine and a member of the neuronal cytokine family that can increase neuronal excitability and induce convulsion (19). Studies have shown that IL-6 levels increase significantly after Flu infection and are correlated with the severity of febrile seizures and brain damage in children (20–23). The results of the present study revealed significantly increased levels of serum IL-6 in the FC group, suggesting that serum IL-6 plays a role in inducing convulsive, which supports the results of the previous study.

The complement system is the center of the immune inflammatory response, helps to eliminate pathogens, and is the first line of defense against pathogenic microbial infection. Complement C3 and C4 are found mainly in plasma and are acute-phase proteins. In the early stage of Flu infection, complement C3 and C4 are activated and released in large quantities, which can stimulate the phagocytosis of immune cells and play an early defense role (24, 25). In the present study, complement C3 and C4 levels were significantly elevated in the FC group, and febrile seizures were likely associated with the activation of complement C3 and C4.

CSF-TP is normally or mildly elevated in children with influenza, especially in FluA-infected CSF, and febrile seizures can occur early in the course of the disease. These patients are prone to complicated febrile seizures (26, 27). The current study revealed that the FluA infection rate and CSF-TP level were significantly greater in the FC group than that in the NFC group. Considering that, after Flu infection in children, the body develops an immune response leading to the release of many inflammatory factors, which reduces vascular permeability. Combined with the imperfect function of the blood‒brain barrier in children, all early Flu infections are prone to result in febrile seizures, which is in agreement with the above studies (28–30).

## Conclusion

In this study, CRP ≥ 7.57 mg/L, IL-6 ≥ 9.84 pg/ml, Days from onset of Flu symptoms to hospitalization ≤4.5 days, CSF-TP ≥ 194.8 mg/L, and FluA were found to be independent risk factors for severe influenza combined with febrile seizures. During treatment, clinicians should closely monitor these indicators, which can help clinicians determine the risk of severe influenza with febrile convulsions. Children with influenza who meet these risk factors should adjust the treatment plan in time and give more active treatment to reduce the incidence of critically ill patients and reduce the occurrence of serious complications.

This was a retrospective study, and the sample size was single-center. In the future studies, further multi-center in-depth research will be conducted to increase innovation.

## Data Availability

The original contributions presented in the study are included in the article/Supplementary Material, further inquiries can be directed to the corresponding author.
